# Leveraging Ethnic Group Incidence Variation to Investigate Genetic Susceptibility to Glioma: A Novel Candidate SNP Approach

**DOI:** 10.3389/fgene.2012.00203

**Published:** 2012-10-12

**Authors:** Daniel I. Jacobs, Kyle M. Walsh, Margaret Wrensch, John Wiencke, Robert Jenkins, Richard S. Houlston, Melissa Bondy, Matthias Simon, Marc Sanson, Konstantinos Gousias, Johannes Schramm, Marianne Labussière, Anna Luisa Di Stefano, H.-Erich Wichmann, Martina Müller-Nurasyid, Stefan Schreiber, Andre Franke, Susanne Moebus, Lewin Eisele, Andrew T. Dewan, Robert Dubrow

**Affiliations:** ^1^Yale School of Public Health, Yale School of MedicineNew Haven, CT, USA; ^2^Department of Epidemiology and Biostatistics, University of California San FranciscoSan Francisco, CA, USA; ^3^Department of Neurological Surgery, University of California San FranciscoSan Francisco, CA, USA; ^4^Laboratory Medicine and Pathology, Mayo Clinic College of MedicineRochester, MN, USA; ^5^Division of Genetics and Epidemiology, Institute of Cancer ResearchSutton, UK; ^6^Dan L. Duncan Cancer Center, Baylor College of MedicineHouston, TX, USA; ^7^Neurochirurgische Universitätsklinik, Universitätskliniken BonnBonn, Germany; ^8^Centre de Recherche de l’Institut du cerveau et de la moelle épinière, Université Pierre et Marie Curie-Paris VIParis, France; ^9^AP-HP, Groupe Hospitalier Pitié-Salpêtrière, Service de Neurologie MazarinParis, France; ^10^Institute of Epidemiology I, Helmholtz Zentrum München – German Research Center for Environmental HealthNeuherberg, Germany; ^11^Institute of Medical Informatics, Biometry and Epidemiology, Ludwig-Maximilians-UniversitätMunich, Germany; ^12^Klinikum GrosshadernMunich, Germany; ^13^Institute of Genetic Epidemiology, Helmholtz Zentrum München – German Research Center for Environmental HealthNeuherberg, Germany; ^14^Department of Medicine I, University Hospital Grosshadern, Ludwig-Maximilians-UniversitätMunich, Germany; ^15^First Medical Department, University Clinic Schleswig-HolsteinKiel, Germany; ^16^Institute of Clinical Molecular Biology, Christian-Albrechts-University KielKiel, Germany; ^17^Institute for Medical Informatics, Biometry and Epidemiology, University Hospital of Essen, University Duisburg-EssenEssen, Germany; ^18^Department of Haematology, University Hospital of Essen, University Duisburg-EssenEssen, Germany

**Keywords:** glioma, candidate SNP association study, ancestry informative markers, admixture, race, ethnicity, brain cancer

## Abstract

**Objectives:** Using a novel candidate SNP approach, we aimed to identify a possible genetic basis for the higher glioma incidence in Whites relative to East Asians and African-Americans. **Methods:**  We hypothesized that genetic regions containing SNPs with extreme differences in allele frequencies across ethnicities are most likely to harbor susceptibility variants. We used International HapMap Project data to identify 3,961 candidate SNPs with the largest allele frequency differences in Whites compared to East Asians and Africans and tested these SNPs for association with glioma risk in a set of White cases and controls. Top SNPs identified in the discovery dataset were tested for association with glioma in five independent replication datasets. **Results:** No SNP achieved statistical significance in either the discovery or replication datasets after accounting for multiple testing or conducting meta-analysis. However, the most strongly associated SNP, rs879471, was found to be in linkage disequilibrium with a previously identified risk SNP, rs6010620, in *RTEL1*. We estimate rs6010620 to account for a glioma incidence rate ratio of 1.34 for Whites relative to East Asians. **Conclusion:** We explored genetic susceptibility to glioma using a novel candidate SNP method which may be applicable to other diseases with appropriate epidemiologic patterns.

## Introduction

Incidence rates of adult primary malignant brain tumors (PMBT), most of which are gliomas (Kohler et al., 2011), vary among ethnic groups (Darefsky and Dubrow, 2009; Dubrow and Darefsky, 2011). The age-standardized incidence rate for northern American non-Hispanic Whites is 2.5–3.0 times the rate among East Asians and around twice the rate among African-Americans. The latter ratio is likely to be higher for comparisons of White to African populations, given the ∼20% European content of the African-American genome (Patterson et al., 2004); however presently there are no data allowing an evaluation (Darefsky and Dubrow, 2009). These ethnic differences in PMBT incidence are unlikely to be solely ascribable to factors such as access to care or diagnostic facilities; in particular, the White-East Asian difference is observed in comparisons among different countries as well as within the United States, where both groups have similar access (Darefsky and Dubrow, 2009; Dubrow and Darefsky, 2011). Notably, ethnic incidence variation has been observed for both grade IV glioma (glioblastoma, or GBM) and non-GBM tumors (Dubrow and Darefsky, 2011).

The only established environmental risk factor for glioma is exposure to high-dose ionizing radiation (Bondy et al., 2008; Ostrom and Barnholtz-Sloan, 2011), which accounts for a small number of cases; furthermore, studies have demonstrated a consistent inverse association with history of allergy (Schoemaker et al., 2010; Lachance et al., 2011) as well as evidence of interaction effects between history of allergy and several established glioma risk alleles (Schoemaker et al., 2010). However, epidemiologic studies have provided no conclusive evidence for diagnostic radiation (Davis et al., 2011), electromagnetic field exposure from residential power lines (Wrensch et al., 1999), smoking (Mandelzweig et al., 2009), alcohol consumption (Efird et al., 2004), nutritional factors (Bondy et al., 2008), or cell phone use (Cardis et al., 2010) as risk factors. Collectively, these observations suggest that ethnic group associated genetic variants, rather than environmental factors, underscore variation in glioma incidence among ethnic groups. Such an assertion is supported by a number of studies suggesting genetic pathways to glioma may differ across ethnicities (Mochizuki et al., 1999; Chen et al., 2001; Das et al., 2002; Wiencke et al., 2005). Following on from this it is possible that the frequencies of haplotypes associated with glioma susceptibility will differ between Whites and East Asians/Africans, such that haplotypes harboring alleles associated with an increased glioma risk would be more prevalent among Whites and conversely haplotypes associated with decreased glioma risk would be more prevalent among East Asians and Africans. Identification of these haplotypes offers the prospect of gaining valuable insight into genes influencing glioma risk.

Here we employed a candidate SNP approach to identify previously unknown genetic variants associated with glioma risk through the identification of SNPs that may tag glioma-related haplotypes. Our primary hypothesis is based on the premise that the same alleles confer protection against glioma in both East Asians and Africans. Consequently, we propose that these alleles are carried at a greater frequency by both East Asians and Africans than by Whites and that genetic regions (i.e., haplotypes) containing SNPs with the greatest allele frequency differences between Whites and both East Asians and Africans (with the same direction of difference) are particularly likely to harbor these alleles. To take into account the possibility that alleles that confer protection in East Asians differ from alleles that confer protection in Africans, we also propose a secondary hypothesis that genetic regions containing SNPs with the greatest allele frequency differences between Whites and either East Asians or Africans, but not both, are likely to harbor protective alleles which are distinct from those identified under the primary hypothesis.

**Figure 1:**
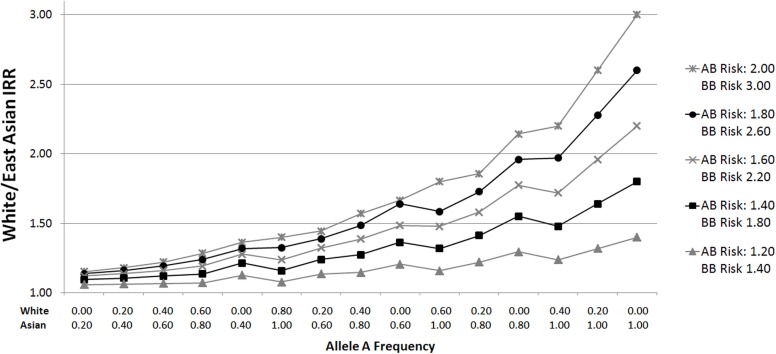
**White/East Asian incidence rate ratios for varying allele distributions and genotypic relative risks**. Plots were generated by calculating incidence rate ratios (IRRs) according to varying genotypic relative risks (GRR) and ethnic group allele frequencies. For example, suppose the GRR for glioma for persons with one B allele is 2.00, and the GRR for persons with two B alleles is 3.00 (relative to those homozygous for the A allele). If the frequency of allele A in Whites is 0.20 (p = 0.2), the proportions of AA (p^2^), AB (2pq), and BB (q^2^) genotypes are 0.04, 0.32, and 0.64, respectively, assuming Hardy Weinberg equilibrium. To calculate a normalized incidence rate, the genotype proportion is multiplied by the associated GRR risk: 0.04 (1.00) + 0.32 (2.00) + 0.64 (3.00) = 2.60. Given an East Asian allele A frequency of 0.80, the East Asian normalized incidence rate is 0.64 (1.00) + 0.32 (2.00) + 0.04 (3.00) = 1.40. The White/East Asian IRR is 1.86 (2.60/1.40) in this scenario. The same calculations apply for White/African IRRs.

Given that ethnic incidence differences are broadly similar for GBM and non-GBM glioma (Dubrow and Darefsky, 2011), we postulate that polymorphisms driving these incidence differences are common across these glioma subtypes, and therefore consider all gliomas combined without stratification. Since large differences in allele frequency are needed to account for even a relatively small portion of the White/East Asian or White/African incidence rate ratio (Figure 1), we restrict our analyses to SNPs showing the largest frequency differences.

## Materials and Methods

### Selection of candidate SNPs

To select candidate SNPs we used allele frequency data on unrelated individuals from six populations included in the International HapMap Project Phase III (Altshuler et al., 2010): 113 Utah residents with Northern and Western European ethnicity (CEU); 102 Toscans from Italy (TSI); 137 Han Chinese from Beijing, China (CHB); 113 Japanese from Tokyo, Japan (JPT); 147 Yoruba from Ibadan, Nigeria (YRI); and 110 Luhya from Webuye, Kenya (LWK). We grouped CEU and TSI together as Whites, CHB and JPT as East Asians, and YRI and LWK as Africans. Data from Phase III, release 28 were downloaded from the HapMap Project File Transfer Protocol^1^. In this release, frequency of genotype missingness per SNP was required to be <0.05 per population, and SNPs were excluded with Hardy Weinberg *P* < 10^−7^. We used in-house Perl scripts to calculate allele frequencies and call rates, according to ethnic group, for SNPs genotyped previously for our discovery genome-wide association study (GWAS) of high-grade adult glioma (Wrensch et al., 2009). SNPs with call rates <95% among Whites, East Asians, or Africans, respectively, were excluded. For each SNP, differences in reference allele frequencies were calculated for Whites vs. East Asians and Whites vs. Africans, as well as the average difference if differences were in the same direction (e.g., the frequency of allele A is low in Whites but high in both East Asians and Africans).

To test our primary hypothesis, we selected SNPs with the greatest average allele frequency differences (provided equivalent directionality) as defined by three categories: “Highest” (≥0.70), “High” (0.60 to <0.70), and “Moderate” (0.40 to <0.60). To test our secondary hypothesis, we selected SNPs for which the allele frequency difference was Highest (≥0.70) in one population comparison, but Low (<0.40) in the other (“Highest/Low”).

### Discovery dataset

Descriptive characteristics of discovery set cases and controls are presented in Table 1. Subjects providing genotype data for the discovery phase included 692 high-grade glioma cases and 3,992 controls originally assembled for the 2009 GWAS of glioma by Wrensch et al. (2009). Briefly, cases included 622 individuals of European ethnicity from the San Francisco Adult Glioma Study (AGS) and 70 from The Cancer Genome Atlas (TCGA; McLendon et al., 2008), aged 20 or older with incident histologically confirmed anaplastic astrocytoma (*n* = 97) or GBM (*n* = 595; International Classification of Diseases for Oncology, morphology codes 9380–9481). Controls included 602 subjects from AGS identified using random digit dialing and frequency matched to cases on age, sex, and ethnicity, as well as 3,390 subjects from the Illumina iControl Database^2^. All subjects were confirmed to be unrelated and of European ethnicity by multidimensional scaling analysis.

**Table 1:** **Discovery set subject characteristics^a^**.

Characteristic^b^	Cases		Controls	
	Adult Glioma Study (*n* = 622)	The Cancer Genome Atlas (*n* = 70)	Adult Glioma Study (*n* = 602)	Illumina iControls (*n* = 3,390)
Age	55 ± 0.5	54 ± 1.7	56 ± 0.6	29 ± 0.4
**GENDER**				
Male	398 (64%)	40 (57%)	319 (53%)	1,254 (37%)
Female	224 (36%)	30 (43%)	283 (47%)	2,136 (63%)
**TUMOR SUBTYPE**				
Grade III	97 (16%)	0 (0%)	–	–
Grade IV	525 (84%)	70 (100%)	–	–

*^a^From Wrensch et al. (2009)*.

*^b^Table values are mean ± standard error for continuous variables and *n* (column%) for categorical variables*.

### Discovery dataset genotyping

Details of sample preparation and genotyping have been provided previously (Wrensch et al., 2009). Briefly, DNA from all AGS cases and controls was isolated from whole blood using Qiagen’s Gentra Puregene DNA isolation kit, and genotyping was conducted using Illumina’s HumanCNV370-Duo BeadChip. AGS samples were required to have a call rate of at least 98%. SNPs deviating from Hardy Weinberg equilibrium in AGS or Illumina controls (*P* < 10^−5^) were excluded from further analysis, as were those with greater than 5% missing data in any of the four subject groups (AGS cases or controls, TCGA cases, Illumina controls).

### Replication datasets

We investigated our top candidate SNPs from the discovery dataset in five independent sets of cases and controls. Detailed procedures of subject selection and genotyping have been described previously (Shete et al., 2009; Wrensch et al., 2009; Sanson et al., 2011). Mayo Clinic cases (*n* = 176), 65% with GBM and 35% with grade III glioma, were diagnosed in Rochester, Minnesota between 2005 and 2008. Controls (*n* = 174) were identified from among individuals who had a general medical exam at the Mayo Clinic, and were matched to cases on sex, age, race, and residence. All cases and controls were genotyped using Illumina Human 610Quad arrays.

The four other replication datasets (UK, US, French, and German) were previously included in a pooled GWAS of glioma (Shete et al., 2009; Sanson et al., 2011). Briefly, the UK GWAS comprised 631 cases ascertained through the INTERPHONE study (Cardis et al., 2010) and 2,699 controls from the 1958 Birth Cohort (Power and Elliott, 2006). The US GWAS comprised 1,247 cases recruited through MD Anderson Cancer Center in Houston, Texas and 2,236 controls from the Cancer Genetic Markers of Susceptibility study (Hunter et al., 2007). The French GWAS comprised 1,423 cases from the Service de Neurologie Mazarin, Groupe Hospitalier Pitié-Salpêtrière Paris, and 1,190 controls from the SU.VI.MAX study (Hercberg et al., 2004). The German GWAS comprised 846 cases recruited from the University of Bonn Medical Center, and 1,310 controls from the KORA (Holle et al., 2005; Wichmann et al., 2005), POPGEN (Krawczak et al., 2006), and Heinz Nixdorf RECALL studies (Schmermund et al., 2002). Cases in the UK and US GWAS were genotyped using Illumina Human 610Quad arrays, and cases from the French and German GWAS were genotyped using Illumina HumanHap660 arrays. Controls in the UK GWAS were genotyped using Illumina Human 1M Duo arrays; the US controls on Illumina HumanHap240, 300, and 500 arrays; the French controls on Illumina HumanHap660 arrays; and the German controls using Illumina HumanHap550 arrays.

### Statistical analyses

Odds ratios and 95% confidence intervals for the association of candidate SNPs with glioma in the discovery and replication sets were calculated using unconditional logistic regression under an additive model (0, 1, or 2 copies of the minor allele). Potential population stratification was adjusted for using principal components derived by the EIGENSTRAT method and included in the logistic regression model (Price et al., 2006).

Discovery set results were evaluated in comparison to Bonferroni-adjusted significance thresholds based on a study-wide significance threshold of 0.05, calculated separately for each of the four subgroups of candidate SNPs [Highest allele frequency difference (≥0.70), High (0.60 to <0.70), Moderate (0.40 to <0.60), and Highest/Low (≥0.70 in one population, <0.40 in the other population)] such that the significance thresholds accounted for the prior probability of association with glioma according to our hypotheses (i.e., *P* = 0.0125 per subgroup). With 38 SNPs in the Highest allele frequency difference category, the significance threshold for this category was 0.0125/38 = 3.29 × 10^−4^. Statistical thresholds for the High, Moderate, and Highest/Low subgroups were 4.70 × 10^−5^, 4.34 × 10^−6^, and 1.61 × 10^−5^, respectively (Table 2). For replication set analyses we used a nominal significance level of 0.05. All *P*-values reported (discovery and replication) are one-sided because of the directionality inherent in the hypothesis being tested. The generic inverse variance method was used (assuming a fixed effects model) to obtain meta-analysis results for combined discovery and replication set data.

**Table 2:** **Number of selected SNPs by allele frequency difference category with corresponding Bonferroni-adjusted significance thresholds**.

	Category	Number of SNPs	Significance threshold
Primary hypothesis	Highest mean allele frequency difference (≥0.70)	38	3.29 × 10^−4^
	High mean allele frequency difference (0.60 to <0.70)	266	4.70 × 10^−5^
	Moderate mean allele frequency difference (0.40 to <0.60)	2,883	4.34 × 10^−6^
Secondary hypothesis	Highest/low allele frequency differences (≥0.70 in one comparison and <0.40 in the other)	774	1.61 × 10^−5^

Analyses were conducted and data visualized using SAS software version 9.2 (SAS Institute, Cary, NC, USA), Haploview version 4.2 (Barrett et al., 2005), R version 2.13.1, Microsoft Excel, Galaxy Project software (Blankenberg et al., 2001), and Review Manager Version 5.1.4 (Cochrane Collaboration, Oxford, UK).

## Results

### Selected candidate SNPs

**Figure 2:**
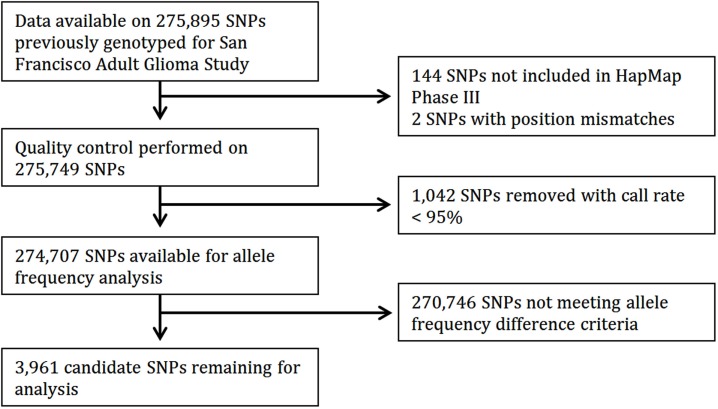
**Flow diagram of candidate SNP selection from HapMap**.

**Figure 3:**
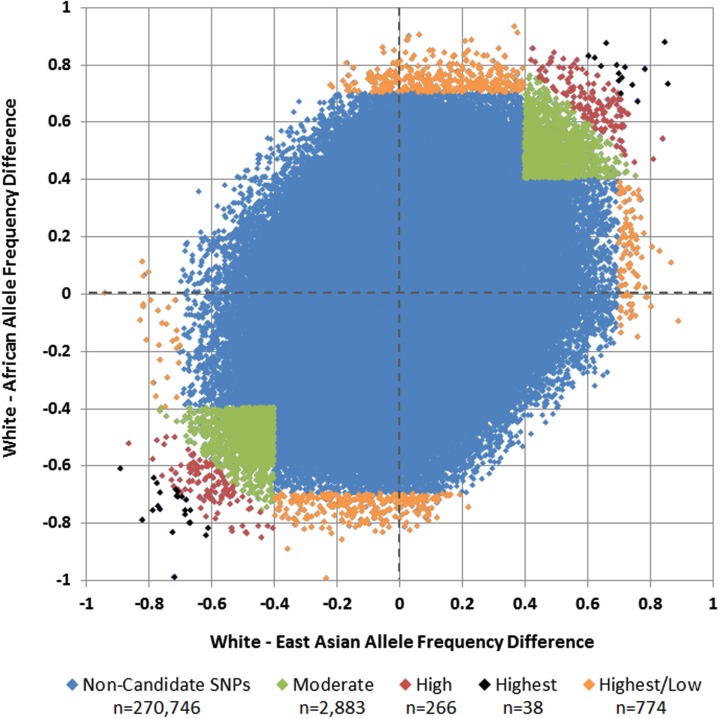
**White vs. African and White vs. East Asian allele frequency differences and selected candidate SNPs**. Each SNP is plotted by its allele frequency difference between Whites and Africans vs. its allele frequency difference between Whites and East Asians. Green SNPs in the upper-right and lower-left quadrants represent those with mean allele frequency differences of 0.40 to <0.60 (Moderate), red SNPs represent those with mean allele frequency differences of 0.60 to <0.70 (High), and black SNPs represent those with mean allele frequency differences ≥0.70 (Highest). Orange SNPs around the perimeter represent those in which the allele frequency difference was at least 0.70 in one comparison, but less than 0.40 in the other (Highest/Low).

Of 275,895 SNPs for which genotype data on discovery glioma cases and controls were previously available, HapMap data were not available or were of insufficient quality for 1,188 (0.43%). Predetermined allele frequency difference criteria were met for 3,961 of the 274,707 remaining SNPs (Figure 2). We identified 2,883 SNPs in the Moderate (0.40 to <0.60) allele frequency difference category, 266 in the High (0.60 to < 0.70) category, 38 in the Highest (≥0.70) category, and 774 in the Highest/Low category (≥0.70 in one population comparison, but <0.40 in the other; Table 2; Figure 3).

### Discovery set

**Figure 4:**
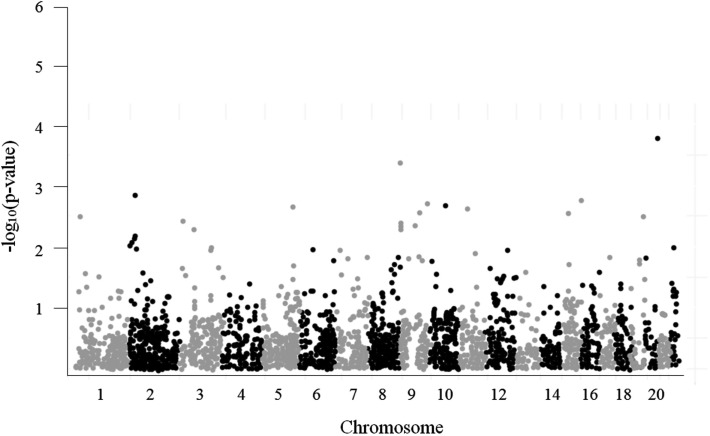
**Manhattan plot for 3,961 tested candidate SNPs**.

A Manhattan plot of the 3,961 SNP-glioma associations is shown in Figure 4. The most strongly associated SNP, rs879471 in *STMN3* on chromosome 20q13 (*P* = 1.72 × 10^−4^), maps 39.9 kb from rs6010620, a SNP intronic to *RTEL1* that was previously identified as a top hit in the GWAS conducted by Wrensch et al. (2009). Conditioning rs879471 on rs6010620 did not, however, provide evidence of a separate signal (*P* = 0.22), so rs879471 was excluded from further analysis.

While no SNP association attained our predetermined Bonferroni-adjusted significance levels (Table 2), 10 genes (*SMARCA2*, *BRE*, *SLCO3A1*, *MORN5*, *C10orf11*, *RBM27*, *PTPRJ*, *SPIB*, *NMNAT1*, and *RPUSD3*) were identified containing at least one SNP with *P* < 0.01. In order to investigate SNPs that may be markers of glioma risk but were excluded by our strict allele frequency criteria, we tested 260 additional SNPs in these 10 genes and within 5 kb upstream and downstream for association with glioma risk (regardless of ethnic allele frequency differences). We excluded four genes (*SLCO3A1*, *C10orf11*, *PTPRJ*, and *NMNAT1*) from further analysis because the direction of association of additional tested SNPs with glioma risk was inconsistent with our hypothesis. All SNPs with *P* < 0.01 across the six remaining genes (*SMARCA2*, *BRE*, *MORN5*, *RBM27*, *SPIB*, and *RPUSD3*) were selected for replication (*n* = 20 SNPs, nine of which were identified in the secondary discovery analysis).

### Replication sets

Discovery and replication set results for the 20 selected SNPs are presented in Table 3. Genotype data were not available for rs4464229 or rs3863 in the UK, US, French, or German sets, and data were not available for rs4666022 in any of the replication sets. For the remaining SNPs, four achieved nominal significance (*P* < 0.05) with an odds ratio in the same direction as in the discovery set in one of the five replication sets and one achieved nominal significance in two of the replication sets; however, none achieved statistical significance in a replication set after correcting for multiple comparisons, and none achieved nominal significance in the meta-analysis.

**Table 3:** **Discovery and replication set results**.

rsID	Gene	Discovery set *P*-value^a^	UCSF discovery set (*n* = 692)^b^	Mayo Clinic (*n* = 176)	UK series (*n* = 631)	US series (*n* = 1,247)	French series (*n* = 1,423)	German series (*n* = 846)	Meta-analysis (*n* = 5,015)^d^	*P*-value for heterogeneity^e^
			**Odds ratio (two-sided 95% confidence interval)**						
rs11127125	BRE	5.75E-03	0.83 (0.71, 0.96)	1.25 (0.87, 1.81)	0.89 (0.77, 1.04)	1.17 (1.03, 1.31)*	1.06 (0.93, 1.21)	1.00 (0.86, 1.17)	0.99 (0.93, 1.05)	0.61
rs4464229	BRE	5.05E-04	0.77 (0.65, 0.90)	1.37 (0.93, 2.01)	NA^c^	NA	NA	NA	0.91 (0.81, 1.02)	0.28
rs4666020	BRE	2.90E-04	0.76 (0.64, 0.89)	1.37 (0.93, 2.02)	0.91 (0.77, 1.07)	1.16 (1.02, 1.31)*	1.04 (0.90, 1.20)	1.02 (0.87, 1.19)	0.98 (0.92, 1.04)	0.41
rs4666022	BRE	3.38E-03	0.82 (0.70, 0.95)	NA	NA	NA	NA	NA	NA	-
rs10175508	BRE	7.40E-04	0.79 (0.68, 0.91)	1.21 (0.84, 1.74)	0.89 (0.77, 1.04)	1.13 (1.01, 1.27)*	1.06 (0.93, 1.21)	1.00 (0.86, 1.16)	0.98 (0.93, 1.04)	0.46
rs10173528	BRE	7.80E-03	0.86 (0.77, 0.97)	1.11 (0.82, 1.50)	0.92 (0.81, 1.05)	1.02 (0.92, 1.13)	1.00 (0.90, 1.12)	1.10 (0.97, 1.24)	0.98 (0.94, 1.03)	0.77
rs10173426	BRE	4.09E-03	0.85 (0.75, 0.96)	1.10 (0.80, 1.50)	0.89 (0.78, 1.01)*	1.00 (0.90, 1.10)	0.97 (0.87, 1.09)	1.07 (0.94, 1.22)	0.98 (0.93, 1.02)	0.83
rs13402525	BRE	5.40E-03	0.85 (0.75, 0.96)	1.08 (0.79, 1.48)	0.89 (0.78, 1.01)*	1.00 (0.90, 1.11)	0.98 (0.87, 1.09)	1.08 (0.95, 1.23)	0.98 (0.93, 1.03)	0.81
rs3863	BRE	9.10E-03	1.15 (1.02, 1.30)	0.87 (0.63, 1.21)	NA	NA	NA	NA	1.03 (0.92, 1.16)	0.57
rs11131194	RPUSD3	1.98E-03	0.84 (0.75, 0.95)	0.73 (0.53, 1.02)*	0.90 (0.79, 1.02)	1.01 (0.91, 1.12)	1.06 (0.95, 1.19)	0.93 (0.82, 1.05)	0.97 (0.92, 1.01)	0.61
rs2279895	RBM27	6.95E-03	1.17 (1.03, 1.33)	0.96 (0.69, 1.34)	1.04 (0.91, 1.19)	1.01 (0.91, 1.12)	1.10 (0.98, 1.25)	0.99 (0.86, 1.13)	1.02 (0.97, 1.08)	0.97
rs11953506	RBM27	7.75E-04	1.23 (1.08, 1.40)	0.86 (0.62, 1.21)	1.04 (0.90, 1.19)	1.03 (0.92, 1.15)	1.10 (0.97, 1.24)	0.97 (0.84, 1.12)	1.02 (0.96, 1.08)	0.89
rs11953090	RBM27	1.12E-03	1.22 (1.07, 1.39)	0.87 (0.62, 1.22)	1.04 (0.90, 1.19)	1.01 (0.91, 1.13)	1.10 (0.97, 1.25)	0.96 (0.84, 1.11)	1.02 (0.96, 1.07)	0.89
rs2304033	RBM27	1.03E-03	1.22 (1.08, 1.39)	0.87 (0.62, 1.22)	1.03 (0.90, 1.19)	1.01 (0.91, 1.13)	1.10 (0.97, 1.25)	0.96 (0.84, 1.11)	1.02 (0.96, 1.07)	0.87
rs13155119	RBM27	1.17E-03	1.22 (1.07, 1.39)	0.87 (0.62, 1.22)	1.04 (0.91, 1.20)	1.01 (0.91, 1.13)	1.10 (0.97, 1.24)	0.97 (0.84, 1.11)	1.02 (0.96, 1.07)	0.90
rs3829070	SMARCA2	2.69E-03	0.76 (0.63, 0.92)	0.72 (0.42, 1.24)	0.91 (0.75, 1.11)	0.93 (0.80, 1.09)	1.15 (0.97, 1.36)	0.93 (0.77, 1.13)	0.95 (0.89, 1.02)	0.69
rs3829072	SMARCA2	2.17E-04	0.71 (0.58, 0.86)	1.00 (0.61, 1.66)	1.07 (0.89, 1.28)	0.87 (0.75, 1.01)*	1.09 (0.92, 1.29)	0.84 (0.69, 1.01)*	0.94 (0.89, 1.01)	0.53
rs10964907	SMARCA2	2.19E-03	0.74 (0.61, 0.91)	0.88 (0.51, 1.52)	1.00 (0.82, 1.22)	0.88 (0.75, 1.04)	1.10 (0.92, 1.31)	0.85 (0.69, 1.04)	0.94 (0.88, 1.01)	0.73
rs870272	MORN5	1.03E-03	0.83 (0.73, 0.93)	1.04 (0.75, 1.44)	0.95 (0.84, 1.08)	0.97 (0.88, 1.07)	0.95 (0.85, 1.06)	0.89 (0.79, 1.01)*	0.96 (0.92, 1.01)	0.93
rs3745516	SPIB	1.60E-03	0.81 (0.70, 0.93)	0.78 (0.54, 1.14)	1.03 (0.89, 1.19)	1.10 (0.98, 1.23)	0.97 (0.85, 1.09)	0.94 (0.82, 1.09)	0.98 (0.93, 1.03)	0.64

*^a^*p*-Values are one-sided*.

*^b^*n* = number of cases*.

*^c^NA, data not available*.

*^d^*n* = 868 for rs4464229 and rs3863*.

*^e^Heterogeneity assessed using Cochran’s Q statistic (Cochran, 1954)*.

*Statistical significance at *P *< 0.05.

## Discussion

Here we have applied a novel candidate SNP method to identify glioma risk alleles, taking advantage of ethnic group differences in glioma incidence. Specifically, we tested the hypothesis that genetic regions containing SNPs with extreme differences in allele frequencies across ethnicities harbor variants that drive the ethnic group variation in glioma incidence. Although in the present study no SNPs from our discovery set reached our predetermined significance thresholds, we identified 10 genes containing one or more SNPs with *P* < 0.01, and selected 20 SNPs across six genes for replication. However, no SNP was statistically significant in any of the replication sets after accounting for multiple comparisons, and no SNP was nominally significant in the meta-analysis.

There were some limitations of this study which may have prevented the detection of glioma-associated SNPs. While our allele frequency difference criteria for candidate SNPs were designed to be inclusive of SNPs that could be responsible for a meaningful risk difference across ethnic groups, it is possible that these criteria excluded SNPs that are, in fact, associated with glioma risk but did not meet our criteria. Additionally, given 692 cases and 3,992 controls in our discovery set, our power calculations demonstrate that for a moderately common putative risk allele (0.20 allele frequency), we had 80% power to detect an odds ratio as low as 1.46. Yet for a relatively rare risk allele (0.05 allele frequency), we had 80% power to detect an odds ratio no lower than 1.88. Thus, it is plausible that our set of candidate SNPs includes one or more variants with low to moderate association with glioma risk, but that we were underpowered to detect such an association. Furthermore, it should be noted that although we postulated that polymorphisms driving ethnic group incidence differences are common across glioma subtypes, it is possible that differences in the glioma subtype distribution in the discovery and replication sets impacted the replicability of our findings. The ability of our approach to detect SNPs that tag glioma-related haplotypes may also have been degraded by heterogeneity across ethnic groups in the haplotype that a given tagging SNP represents. Finally, we note that our study was unable to assess potential interaction effects between risk loci, gene-environment interactions, or the role of rare variants.

Although this study did not lead to the discovery of novel glioma-associated SNPs, it is noteworthy that our most strongly associated candidate SNP, rs879471, was in strong linkage disequilibrium with rs6010620 (*D*′ = 0.78 in HapMap CEU + TSI, data from Haploview version 4.2), a top hit from the Wrensch et al. (2009) GWAS. This suggests the successful identification of a haplotype that differs in frequency across ethnic groups and is related to glioma risk. In this respect the A allele of rs6010620, which is protective against glioma (OR = 0.68, 95% CI: 0.58–0.79), is considerably more common in East Asians than Whites (frequency of 0.697 vs. 0.228, respectively), but did not meet our strict allele frequency difference criteria because the frequency in Africans was 0.019. On the basis of a calculation similar to that presented in Figure 1, we would conclude that this SNP is sufficient to account for an incidence rate ratio of 1.34 for Whites relative to East Asians. Notably, another top hit from the same GWAS, rs1412829, has a risk allele C (OR = 1.39, 95% CI: 1.24–1.57) that is more common in Whites than East Asians or Africans but also did not meet our allele frequency criteria (frequency of 0.402, 0.104, and 0.009, respectively). Based on our calculations, this SNP can account for a White to East Asian incidence rate ratio of 1.22, and a White to African incidence rate ratio of 1.30. Thus, these two SNPs alone may account for a meaningful proportion of the observed inter-ethnic incidence rate ratios. None of the other five established glioma susceptibility loci contribute to the inter-ethnic incidence rate differences (Table 4).

**Table 4:** **Contribution to ethnic incidence rate ratios by established glioma susceptibility loci**.

SNP	Reference allele	Reference allele frequency			Odds ratio (95% confidence interval)^a^	White-East Asian incidence rate ratio	White-African incidence rate ratio
		White	East Asian	African	
rs6010620	A	0.23	0.70	0.02	0.68 (0.58, 0.79)^b^	1.34	0.90
rs1412829	C	0.40	0.10	0.01	1.39 (1.24, 1.57)^b^	1.22	1.30
rs2736100	G	0.54	0.41	0.42	1.27 (1.19, 1.37)^c^	1.05	1.05
rs4295627	G	0.15	0.24	0.16	1.36 (1.29, 1.43)^c^	0.95	0.99
rs4977756	G	0.37	0.23	0.34	1.24 (1.19, 1.30)^c^	1.06	1.01
rs498872	T	0.28	0.24	0.08	1.18 (1.13, 1.24)^c^	1.01	1.07
rs2252586	T	0.28	0.02	0.32	1.18 (1.11, 1.25)^d^	1.09	0.99

*^a^Odds ratios were calculated according to an additive model (0, 1, or 2 copies of the reference allele)*.

*^b^Wrensch et al. (2009)*.

*^c^Shete et al. (2009)*.

*^d^Sanson et al. (2011)*.

The candidate SNP approach used in this study provides a viable alternative to admixture mapping, which investigates the genetic makeup of recently admixed groups to localize disease-related variants (Patterson et al., 2004; Smith and O’Brien, 2005). In the context of glioma research, the admixture method would ideally be applied to a set of African-American cases and controls, where excess European ethnicity shared among African-American cases would be suggestive of genetic regions that may play a role in glioma risk. For conditions like hypertension, where prevalence differs widely between ethnicities and samples from an admixed group are available, the pairing of admixture mapping with GWAS has proven to be an effective means of identifying disease-related variants (Levy et al., 2009; Zhu et al., 2011). However, when samples from an admixed group are not available, we present our approach as an alternative complement to GWAS. Specifically, this approach to identify disease-causing variants is attractive where (1) the disease incidence differs substantially across two or more ethnic groups; (2) ethnic group differences tend to persist independent of geographic location, suggesting a genetic etiology; (3) there is an availability of cases and controls of uniform ethnicity; and (4) an admixture approach is not feasible given the unavailability of a sufficient number of appropriate admixed cases.

While we did not identify novel glioma susceptibility variants in this analysis, we conclude that the additional risk in White populations conferred by rs6010620 and rs1412829 lends support to our initial hypothesis, and provides an impetus for a larger discovery set and/or pursuing admixture mapping. Given the rarity of glioma among African-Americans and the resultant difficulties inherent in collecting enough African-American cases to perform an admixture mapping study, further application of our method may be the preferred approach.

## Conflict of Interest Statement

The authors declare that the research was conducted in the absence of any commercial or financial relationships that could be construed as a potential conflict of interest.

## Footnotes

^1^ftp://ftp.ncbi.nlm.nih.gov/hapmap

^2^http://www.illumina.com/science/icontroldb.ilmn
